# Establishment of Transgenesis in the Demosponge *Suberites domuncula*

**DOI:** 10.1534/genetics.118.301121

**Published:** 2018-08-16

**Authors:** Roger Revilla-i-Domingo, Clara Schmidt, Clara Zifko, Florian Raible

**Affiliations:** * Max F. Perutz Laboratories, University of Vienna, Vienna BioCenter, 1030, Austria; † Research Platform “Rhythms of Life”, University of Vienna, Vienna BioCenter, 1030, Austria

**Keywords:** Porifera, sponges, transgenics, *Suberites domuncula*

## Abstract

Sponges (Porifera) represent one of the most basally branching animal clades with key relevance for evolutionary studies, stem cell biology, and development. Despite a long history of sponges as experimental model systems, however, functional molecular studies are still very difficult to perform in these animals. Here, we report the establishment of transgenic technology as a basic and versatile experimental tool for sponge research. We demonstrate that slice explants of the demosponge *Suberites domuncula* regenerate functional sponge tissue and can be cultured for extended periods of time, providing easy experimental access under controlled conditions. We further show that an engineered expression construct driving the *enhanced green fluorescence protein* (*egfp*) gene under control of the *Suberites domuncula β-actin* locus can be transfected into such tissue cultures, and that faithfully spliced transcripts are produced from such transfected DNA. Finally, by combining fluorescence-activated cell sorting (FACS) with quantitative PCR, we validate that transfected cells can be specifically reisolated from tissue based on their fluorescence. Although the number of detected enhanced green fluorescent protein (EGFP)-expressing cells is still limited, our approach represents the first successful introduction and expression of exogenous DNA in a sponge. These results represent a significant advance for the use of transgenic technology in a cornerstone phylum, for instance for the use in lineage tracing experiments.

SPONGES (Porifera) are morphologically simple filter-feeding animals, lacking muscles, organs, and a nervous system. Molecular phylogeny consistently places them among the most basally branching metazoan phyla (Philippe *et al.* 2009, 2011; Ryan *et al.* 2013; Feuda *et al.* 2017). Sponges are, therefore, a critical group in the search for the origins of metazoan multicellular processes and in understanding the evolution of the nervous system (Srivastava *et al.* 2010; Ereskovsky *et al.* 2012; Richter and King 2013; Fortunato *et al.* 2014; Leys 2015; Adamska 2016; Kenny *et al.* 2018). Their morphological simplicity, together with their remarkable regeneration capacity, makes them an attractive model for regeneration and stem cell biology (Simpson 1984; Funayama 2013, 2018; Alié *et al.* 2015). In addition, sponges harbor dense and diverse communities of symbiotic bacteria, making them an important model for the study of metazoan host–microorganism interactions (Hentschel *et al.* 2012; Pita *et al.* 2016).

Even though sponges have a long history as experimental model systems and hold strong potential for advancing our understanding of evolutionary, developmental, and stem cell biology, mechanistic molecular insights into this important clade have remained limited. Whereas silencing of sponge genes by double-stranded RNA (dsRNA) interference yields promising, but variable results (Rivera *et al.* 2011, 2013; Windsor Reid *et al.* 2018), genome manipulation techniques are not yet established in any sponge species. Likewise, robust methods to trace cell lineages are currently lacking, making it difficult to reliably characterize stem cells and their differentiation potential at the cellular level. A major challenge in establishing more versatile functional techniques is the difficulty in introducing genetic material into sponge cells to allow for exogenous gene expression (Adamska 2016). To remove this important obstacle, we have developed a robust method to introduce exogenous expression constructs into sponge cells. Our approach takes advantage of a reliable explant culture system that we have established in the demosponge *Suberites domuncula*, a species that can easily be maintained in the lab. Targeted genome amplification and molecular cloning have allowed us to engineer an expression construct in which an *enhanced green fluorescence protein* (*egfp*) gene is placed under control of the endogenous *β-actin* locus of *S. domuncula*. Finally, we have established conditions for a robust polyethyleneimine-based transfection protocol. To our knowledge, this study represents the first proof of principle of transgenesis in sponges. Although the rate of transgenesis is still low, it will already be suitable for some applications, such as lineage tracing, while serving as a promising basis for the establishment of further molecular functional techniques, and thus add a versatile tool for sponge research.

## Materials and Methods

### *S. domuncula* collection and maintenance

*S. domuncula* specimens were collected from Punta Sutile (Muggia, Italy) at a depth of 10–15 m. Animals were maintained in 200-liter closed seawater aquaria, as described in the extended Materials and Methods in Supplemental Material, File S1.

### *S. domuncula* explant culture

Explants of ∼20 × 10 × 2 mm were cut from adult *S. domuncula* specimens using sterile razor blades and placed in a culture vessel (or, alternatively, on glass slides that were placed inside a culture vessel) with sterile seawater. Sterile seawater was prepared by filtering aquaria seawater through a 0.22-µm polyethersulfone filter membrane (TPP, Switzerland). Explants were left unperturbed at ∼15° for 2–4 days and then transferred to the aquaria, where they were fed according to the same feeding regime as the adult specimens (see extended Materials and Methods in File S1). Observations were made through a Leica MZ 16 FA microscope (Leica Microsystems), and images were taken with a Leica DFC 300FX camera (Leica Microsystems).

### Identification and characterization of the *S. domuncula β-actin* gene homolog

The *S. domuncula* transcriptome was sequenced and *de novo* assembled as described in extended Materials and Methods in File S1. The assembled transcriptome was used to identify the *Suberites domuncula β-actin* gene homolog, and the *β-actin* gene locus was characterized as described in extended Materials and Methods in File S1.

### Generation of EGFP reporter construct

Fusion-PCR (Wang *et al.* 2002) was used to combine genomic sequences of the *β-actin* gene locus with the *egfp* sequence codon-optimized for *Hydra* according to the scheme of Figure 2A. The PCR product was cloned into the PGEM-T vector (Promega). Plasmids from positive clones were purified using the QIAprep Spin Miniprep Kit (Qiagen).

### Transfection of the EGFP reporter construct

Small explants of ∼5 × 5 × 1 mm were placed in individual wells of a sterile 24-well plate (CytoOne) with 2 ml of sterile seawater and incubated for 1 hr at room temperature. The transfection reagent jetPEI (Polyplus-Transfection S.A., France) was used according to manufacturer’s guidelines to prepare 200 µl of jetPEI/DNA complex per sample, containing 12 µl of jetPEI reagent and 4 µg of the EGFP reporter DNA plasmid. These 200 μl were added to each explant. After an incubation of 3–4 hr at room temperature, explants were cultured at 15° for 48 hr. They were then either processed for *egfp* DNA/messenger RNA (mRNA) quantification or transferred to a sterile 60-mm cell culture dish (Greiner Bio-One) with sterile seawater and cultured for up to 28 days at 15°. Half of the sterile seawater was replaced with fresh sterile seawater twice a week.

### Quantification of *egfp* DNA and mRNA levels

At the desired stage, explants were lysed in 0.7 ml of buffer RLT Plus (Qiagen). Lysates were vortexed until all tissue became invisible.

Extraction of genomic DNA for *egfp* quantification was performed as described in extended Materials and Methods in File S1. The number of EGFP reporter DNA molecules per cell was measured by quantifying the number of *egfp* molecules relative to the single-copy gene *g7a* by qPCR (for identification and validation of *g7a*, as well as basic qPCR methodology, see extended Materials and Methods in File S1). We also confirmed that a separate single-copy gene, *rpl11* (transcript ID comp110873_c0_seq1), shows a similar number of DNA copies to *g7a*, indicating that our results do not depend on the choice of a particular single-copy gene (data not shown).

Total RNA for *egfp* mRNA quantification was isolated from lysates ensuring complete removal of genomic DNA (see extended Materials and Methods in File S1). Complementary DNA (cDNA) was synthetized using the QuantiTect Reverse Transcription kit (Qiagen) according to manufacturer’s guidelines. Quantification of *egfp* mRNA was again performed by comparing with the reference gene *g7a* (see extended Materials and Methods in File S1).

### Fluorescence-activated cell sorting (FACS)

To FAC-sort EGFP+ cells, explants were transfected for 48 hr as described above and then cultured for 7 days in 6-mm petri dishes with sterile sea water to allow accumulation of EGFP. Negative control samples (not transfected) were cultured in the exact same conditions. Explants were dissociated into single-cell suspensions, and cell suspensions were stained with Vybrant DNA DyeCycle as described in extended Materials and Methods in File S1. Stained cell suspensions from explants that had been transfected with the EGFP reporter construct (transfected samples) and from explants that had not been transfected (negative control) were analyzed on a FACSARIA IIIu Fluorescence-Activated Cell Sorter (BD Biosciences) as described in extended Materials and Methods in File S1. Cells in the EGFP+ gate of the transfected samples were isolated and their *egfp* mRNA relative to a reference gene quantified as described in extended Materials and Methods in File S1. *egfp* mRNA was also quantified in unsorted aliquots of the same transfected samples.

### Microscopic observation of FAC-sorted cells

To FAC-sort EGFP+ cells for microscopic observations, the FACS-flow of the FACS machine was substituted with filtered seawater. Cells from the EGFP+ gate (EGFP+) or from outside of the EGFP+ gate (EGFP−), were FAC-sorted directly into the wells of a glass-bottom 96-well plate (#655891; Grenier Bio-One) containing 50 µl of sterile seawater. The 96-well plate was kept at 4° during sorting, and stored on ice after sorting was completed. Cells were imaged on an inverted Observer Z1 microscope (Zeiss) using the eGFP filter set (BP 470/40, FT 495, BP 525/50) and the DAPI filter set (G 365, FT 395, BP 445/50), and images were taken using a pco. 1600 camera (PCO AG). All cells were imaged under the same settings, to allow comparison. The ImageJ software was used to generate overlays.

### Data availability

The described expression construct is available upon request. File S1 contains the extended Materials and Methods including additional references for this part and Figures S1–S5. File S2 contains the assembled *S. domuncula* transcriptome in multi-fasta format. Figure S1 provides schemes of the sexual and asexual life cycle of *S. domuncula*. Figure S2 provides a survival curve indicating that *S. domuncula* slice explants can regenerate and survive for months in controlled culture conditions. Figure S3 shows a systematic analysis of codon usage in *S. domuncula* and a comparison of the usage of rare sponge codons in two different codon-optimized EGFP variants. Figure S4 provides an overview of how the absolute number of mRNA molecules per cell were estimated using internal controls. Figure S5 provides molecular phylogenetic support for the identity of *S. domuncula* Vasa and two other DEAD box helicases identified in the course of this study. Supplemental material available at Figshare: https://doi.org/10.25386/genetics.6974267.

## Results

### Establishment of an explant regeneration and culture system suitable for transfection experiments

**Figure 1:**
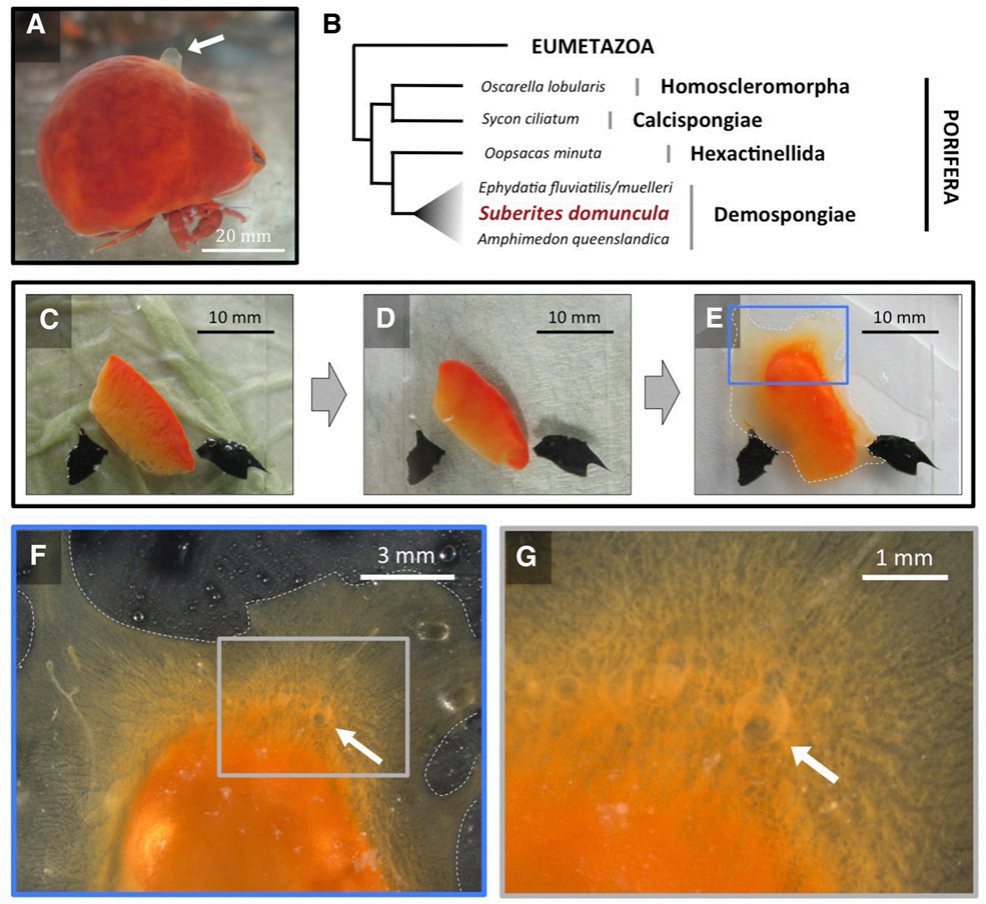
A *Suberites domuncula* explant culture system faithfully recapitulates regeneration. (A) Typical adult *S. domuncula* specimen in aquarium. Sponges of the *S. domuncula* species grow on gastropod shells (not visible in this image), which are typically inhabited by a *Paguristes* hermit crab (lower part of the image). (B) Phylogeny of sponges, simplified from Philippe *et al.* (2009), showing the phylogenetic relationship between the four classes of sponges, including selected sponge laboratory model species. (C) Explant of adult specimen placed on a glass slide, immediately after cutting. The explant is immobilized from the tip by a coverslip attached to the glass slide with silicone (black marks). (D) Same explant, 2 days after cutting. (E) Same explant, 14 days after cutting. (F) Higher magnification view of boxed area of the explant shown in E. (G) Higher magnification view of boxed area of the explant shown in F. The white arrow in A, F, and G shows the oscule. The dotted white line in E and F shows the contour of the tissue that has grown attached to the glass slide.

The availability of specimens around the year, and the ability to grow them for extended times under standardized laboratory conditions, are important criteria for the choice of a successful laboratory model. We decided to focus our efforts on *S. domuncula*, a marine sponge broadly distributed in the Mediterranean Sea and the Northern Atlantic and Pacific coasts (Burton 1953; WoRMS Editorial Board 2018). Adult *S. domuncula* specimens can be easily maintained in close seawater aquaria (Le Pennec *et al.* 2003), facilitating experimental access to genetically defined individuals. Adults have a typical size of a few centimeters (Figure 1A) and display typical anatomical features of sponges, including the oscule (Figure 1A arrowhead), *i.e.*, the opening through which the water circulating through the sponge canal system flows out of the animal during feeding. *S. domuncula* possesses a prototypical, marine life style, and belongs to the major class of sponges, the Demospongiae, which covers >90% of sponge species (Funayama 2018), including the marine sponge *Amphimedon queenslandica*, and the freshwater sponges *Ephydatia fluviatilis* and *Ephydatia muelleri* (Figure 1B). Also, *S. domuncula* displays the typical cellular composition of demosponges (Simpson 1984; Funayama 2018), including the two cell types suggested to function as stem cells in sponges: the archeocytes, proposed to be totipotent stem cells, and the choanocytes, suggested to be pluripotent (Funayama 2013). While archeocyte-like cells have been reported in hexactinellids, it is thought that this cell type is completely missing in calcisponges and homoscleromorphs, in which choanocytes are thought to take a totipotent stem cell role (reviewed by Funayama (2018)). *S. domuncula* individuals can reproduce sexually or asexually through gemmules (Figure S1). Reproduction through gemmules has been reported in a number of demosponge species, including *E. fluviatilis* and *E. muelleri*, but not in *A. queenslandica* or nondemosponges (Simpson 1984).

To facilitate the possibility to carry out transfection experiments with this sponge species, we next investigated suitable culture systems. Whereas reaggregates of dissociated cells – so-called primmorphs – have been described for *S. domuncula*, these structures do not show the typical morphology of adult specimens (Custodio *et al.* 1998), possibly limiting the extent of biological features that can be studied. We therefore explored whether *S. domuncula* slice explants could be grown on conventional culture vessels (Figure 1, C–G). To this end, we cut small explants of adult sponge tissue –devoid of the oscula – and placed them in seawater on glass slides, petri dishes, or conventional culture multi-well plates (Figure 1C). After ∼2 days of culture, such explants exhibit a noticeably remodeled shape and attach to the substrate (Figure 1D). After 2 weeks, explants exhibit marked outgrowth in all directions (Figure 1, E and F), consistent with the notion that the slice undergoes regeneration into a functional sponge. Indeed, detailed microscopic observations show that the outgrown structures contain multiple canals that converge onto a miniature oscule (Figure 1, F and G). Live inspection of samples has allowed us to observe outward flow of water through this structure (data not shown), and confirm that the sponge tissue regenerating from such slice explants remains viable for several months (Figure S2). Together, these experiments establish that *S. domuncula* explants regenerate into functional sponges and can be maintained for the long term in culture, providing a useful setup to perform transfection experiments in a biologically meaningful setting.

### Generation and introduction of an EGFP reporter construct into sponge tissue

**Figure 2:**
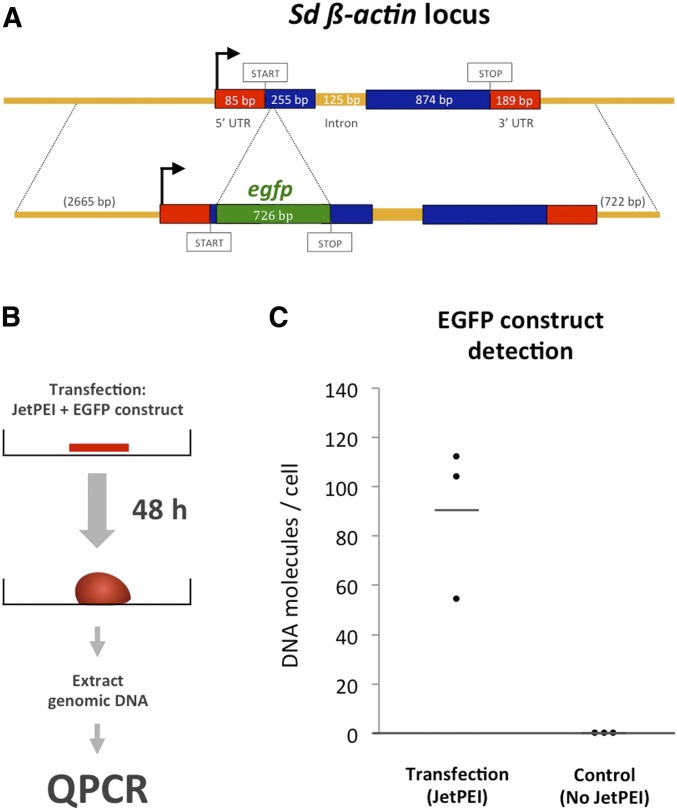
Generation and introduction of an EGFP reporter construct into sponge cells. (A) Structure of the *Suberites domuncula β-actin* gene locus (upper line) and structure of the EGFP reporter construct generated (lower line). (B) Schematic representation of the procedure for transfection and detection of the EGFP reporter construct in sponge cells. (C) Group scatter plot showing the levels of *egfp* reporter construct molecules detected per cell in samples transfected with jetPEI and in controls. Controls were processed exactly as the transfected samples except that jetPEI reagent was not added to the culture medium. Each dot represents an independent transfection experiment. Horizontal bar shows the average of all replicas.

Expression constructs that are based on ubiquitously expressed genes have been helpful in developing transgenesis in various clades (Sun *et al.* 2005; Wittlieb *et al.* 2006; Backfisch *et al.* 2013; Wudarski *et al.* 2017). We therefore decided to generate an expression construct based on the *S. domuncula β-actin* gene (transcript ID comp60760_c0_seq1) that is expressed at high levels in all cells (R. Revilla-i-Domingo and F. Raible unpublished data). As a reporter, we chose an *egfp* gene used in *Hydra* (Wittlieb *et al.* 2006), minimizing the number of codons that are rarely used in *S. domuncula* (Figure S3). The resulting construct (Figure 2A) contains the *egfp* gene inserted after the third codon of the *β-actin* coding sequence. Apart from the insertion of the *egfp* coding sequence, the construct preserves the endogenous structure of the *β-actin* gene locus, including ∼2.7 kb of genomic sequence upstream of the 5′ UTR, ∼0.7 kb downstream of the 3′UTR, and an intron of 125 bp (Figure 2A) that we later used to assess splicing of the generated transcript.

Having established this expression construct, we next used it to assess whether DNA could be transfected into the slice explants we established (see Figure 2B). The method we describe here takes advantage of linear polyethyleneimine (jetPEI) as a transfection reagent. This reagent has previously been successfully employed in other marine animals (Lu and Sun 2005; Sun *et al.* 2005), and shown to be more efficient than other typical transfection reagents in a number of systems (Calderon and Sun 2003; Sun *et al.* 2005; Yamano *et al.* 2010; Han *et al.* 2015). To parallelize the transfection assays, sponge explants were grown for 48 hr in multi-well plates in the presence of jetPEI transfection reagent and EGFP reporter construct. To test for introduction of the construct into the cells of the explants, we extracted genomic DNA and measured the number of *egfp* amplicons relative to amplicons of the gene *g7a*/*valyl-tRNA synthetase* (*vars*) (transcript ID comp115870_c0_seq1) by quantitative PCR (qPCR) (Figure 2B). *G7a/vars* is a single-copy gene (Huerta-Cepas *et al.* 2016; extended Materials and Methods in File S1), allowing us to calculate the number of EGFP reporter construct molecules per cell. We consistently detected between 50 and 110 copies of the reporter construct per cell (Figure 2C), indicative of a robust transfection rate. To rule out the possibility that the *egfp* molecules detected were picked up from the culture medium, we also measured the levels of *egfp* in control samples that had been grown and processed exactly as our transfected samples, except that the jetPEI transfection reagent had been omitted from the culture medium. *egfp* was almost undetectable in these control samples (Figure 2C), indicating that the EGFP reporter construct molecules are indeed transfected into sponge cells in the presence of jetPEI.

### Stable expression of faithfully spliced transcripts from transfected sponge tissue

**Figure 3:**
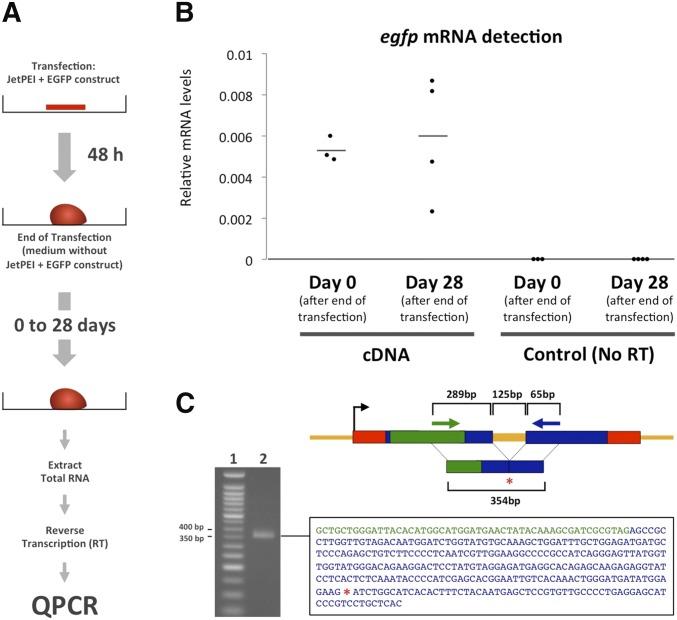
Stable expression of the correctly spliced reporter gene. (A) Schematic representation of the procedure for transfection and detection of *egfp* mRNA. (B) Group scatter plot showing *egfp* mRNA levels relative to a reference gene in cDNA samples from transfected explants and in control (“No RT”) samples. Control samples were processed exactly as cDNA samples except that the reverse transcription enzyme was not added to the reverse transcription reaction. Each dot represents an independent transfection experiment. Horizontal bars show the average of all replicas. (C) The schematic diagram represents the EGFP reporter construct with the position of the primers used to confirm splicing of the intron. The green arrow indicates the position of the left primer, which targets the *egfp* sequence. The blue arrow indicates the position of the right primer, which targets the *β-actin* transcript sequence. The red asterisk indicates the position where the intron was before splicing. The gel electrophoresis image shows a 50-bp DNA ladder (lane 1) and the product of a PCR using the primers shown in the schematic diagram (lane 2). The size of the band in lane 2 is consistent with that expected if the intron has been spliced out. The black rectangle shows the sequence of the PCR product visualized in lane 2 of the gel. Green font indicates that the sequence matches the *egfp* gene, blue font indicates that the sequence matches the *β-actin* transcript, and the red asterisk indicates that the sequence of the intron is missing.

The fact that DNA is taken up by tissue does not warrant that this DNA is accessible to the transcriptional machinery of the host cell. To assess whether the EGFP reporter construct introduced into sponge cells is actually transcribed, we measured relative *egfp* mRNA levels in samples that had been transfected with the EGFP reporter construct for 48 hr (Figure 3). For this, we extracted total RNA from explants immediately after transfection, generated cDNA by reverse transcription (RT), and used qPCR to measure the levels of *egfp* cDNA molecules relative to the aforementioned reference gene *g7a* (Figure 3A), typically expressed ubiquitously in all animal cell types (Huerta-Cepas *et al.* 2016; extended Materials and Methods in File S1), and whose expression level is at ∼230 mRNA molecules per cell in *S. domuncula* (Figure S4). QPCR allowed us to find detectable amounts of *egfp* cDNA molecules in the transfected explants (Figure 3B, “cDNA – Day 0”). To rule out the possibility that carry-over of genomic DNA during total RNA extraction could affect our results, we also measured the levels of *egfp* in control samples, in which the RT step had been omitted. No *egfp* molecules were detected in these control samples (Figure 3B, “Control (NoRT) – Day 0”). We also confirmed that no *egfp* is detected in cDNA samples from explants that had not been transfected with the EGFP reporter construct (data not shown). Overall, these results demonstrate that after 48 hr of transfection, mRNA has been transcribed from our EGFP reporter construct in sponge cells.

To assess if the observed *egfp* transcripts just represented a transient peak, or if our transfection method allowed for the expression of genes for prolonged periods, we next quantified *egfp* mRNA levels in samples that had been cultured for up to 28 days following transfection. We found that the relative *egfp* mRNA levels did not change significantly during these 28 days of culture (Figure 3B, “Day 28”), consistent with *egfp* RNA being transcribed from stably transfected DNA. The high variability in the levels of *egfp* mRNA detected 28 days after transfection may reflect different rates of proliferation of the transfected cells in the four independent biological replicates.

Finally, we took advantage of the presence of an intron in our construct to test whether the transcripts generated from our EGFP reporter construct were properly processed (Figure 3C). For this experiment, we performed a PCR reaction on cDNA samples from transfected explants using primers flanking the intron, with the forward primer residing in the *egfp* coding sequence, and the reverse primer located in the *β-actin* coding sequence downstream of the intron. The resulting amplicon revealed that the intron was precisely spliced out from the original sequence (Figure 3C). Together, these results demonstrate that upon transfection, exogenous DNA can be transcribed and correctly spliced by sponge cells for extended periods. Whereas we presently do not know the exact mechanism by which maintenance of exogenous DNA transcription is achieved (*e.g.*, integration of the exogenous DNA into the sponge genome, maintenance as extrachromosomal arrays, or other), our results are consistent with stable transfection.

### Detection of EGFP fluorescence

**Figure 4:**
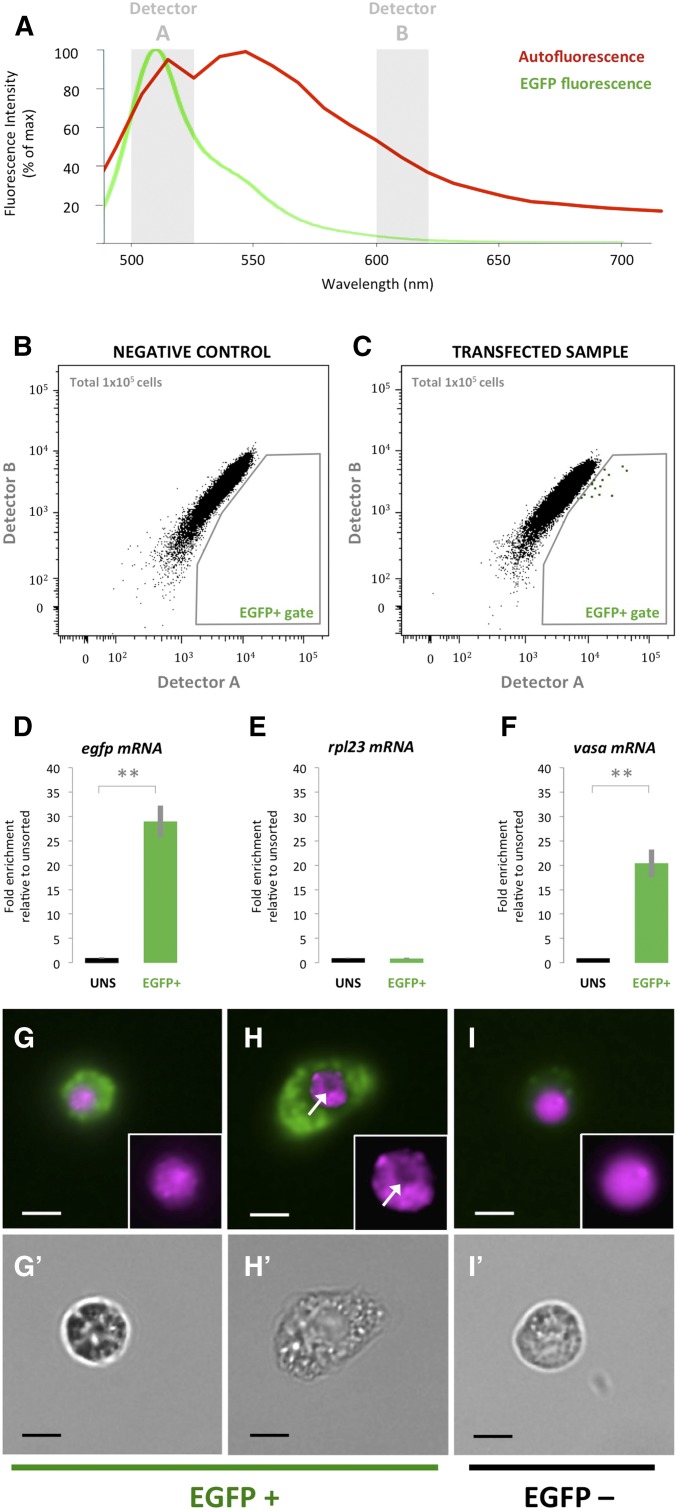
Detection of EGFP fluorescence in sponge cells. (A) Representative autofluorescence spectrum of cells from a typical *Suberites domuncula* sponge under a 488-nm excitation laser (red line) and EGFP fluorescence spectrum (green line; obtained from the BD Biosciences Spectrum Viewer – www.bdbiosciences.com/eu/s/spectrumviewer). The two gray shadings indicate the wavelengths selected in detector A (500–526 nm) and detector B (600–620 nm) to identify the EGFP+ cells. (B) FACS plot showing fluorescence intensity measured by detector A *vs.* detector B in a negative control sample (not transfected). The negative control sample is used as an autofluorescence baseline to draw the EGFP+ gate. (C) FACS plot showing fluorescence intensity measured by detector A *vs.* detector B in a transfected sample. (D–F) Barplots showing the fold enrichment in *egfp* (D), *rpl23* (E), and *vasa* (F) mRNA levels in FAC-sorted EGFP+ cells relative to Unsorted cells. FAC-sorted EGFP+ cells and Unsorted cells were both taken from the same transfected sample. Error bars indicate the SD. The statistical significance was calculated by a nonpaired, one-tailed Student’s *t*-test (*n* = 3). (G–I) Fluorescence images of FAC-sorted cells from the EGFP+ gate (G and H) and from outside of the EGFP+ gate (I). Green shows EGFP fluorescence, and magenta shows Vybrant DNA DyeCycle staining, which stains the nucleus. The white arrow indicates a clear area in the nucleus, consistent with a nucleolus (Funayama 2013). The inserts in G–I show a magnified image of the Vybrant DNA DyeCycle staining for the respective fluorescence image. (G’–I’) White field images of the cells shown in G–I, respectively. All bars, 3 µm.

To test whether the transfection of our EGFP reporter construct results in detectable EGFP fluorescence, we decided to employ FACS, a method that allows rapid, reliable and sensitive detection of fluorescent cells (Figure 4).

Since autofluorescence could potentially affect our capacity to detect EGFP fluorescence, we first analyzed the autofluorescence spectrum of sponge cells illuminated by a 488-nm laser using a confocal microscope, and compared it to the known fluorescence spectrum of EGFP (Figure 4A). To unambiguously distinguish EGFP fluorescence from autofluorescence during FACS, cells were analyzed using a 488-nm laser source with two fluorescence detectors: “Detector A” measured fluorescence in the range of 500–526 nm, coinciding with the peak of the EGFP fluorescence spectrum (Figure 4A), whereas “Detector B” measured fluorescence in the range of 600–620 nm, where the autofluorescence spectrum is still relatively high, while the EGFP fluorescence spectrum is strongly reduced (Figure 4A). Analysis of the cells of a negative control sample (not transfected) with these two fluorescence detectors provided us with an autofluorescence baseline (Figure 4B). We thus expected cells with true EGFP fluorescence to show a shift from this baseline in the “Detector A” axis.

When analyzing tissue transfected with the EGFP reporter, most cells fall in a similar position on the FACS plot to the cells of the negative control (Figure 4C). In contrast to the negative control sample, however, we reproducibly detect a few cells (∼1 in 7000) with a distinct shift from the autofluorescence baseline that is consistent with the presence of EGFP fluorescence in these cells (Figure 4C, “EGFP+ gate”).

To test whether the cells in the “EGFP+ gate” indeed express the EGFP reporter, we FAC-sorted cells from this gate, and measured the relative levels of *egfp* mRNA. We also measured relative *egfp* mRNA levels in unsorted cells of the same transfected sample. The relative *egfp* mRNA levels in the unsorted cells was consistent with those reported in Figure 3B (0.0059 ± 0.0007 relative to our reference gene). In contrast, the relative *egfp* mRNA levels in the cells of the EGFP+ gate were ∼30-fold higher (Figure 4D). As expected, the relative levels of a separate housekeeping gene (*rpl23/COG0093*, transcript ID comp110858_c0_seq1) did not change between the two populations (Figure 4E). These data are consistent with the fact that EGFP is robustly expressed in only a few cells, even though there might be additional cells in which weaker EGFP fluorescence might be masked by the general autofluorescence level. Because our reference gene is expressed at ∼230 mRNA molecules per cell (Figure S4), the results presented in Figure 4D indicate that *egfp* is expressed at ∼40 mRNA molecules per EGFP+ cell.

A final question we addressed was whether the isolated fluorescent cells provided any hint on the nature of the cells accessible to transfection. For this, we investigated FAC-sorted cells by fluorescence microscopy (Figure 4, G–I) and observed cells of a wide range of sizes (for examples see Figure 4, G and H). Our expectation was that mitotically active cells might be more likely to become stably expressing cells, as exogenous DNA should have easier access to the nuclei reforming after mitosis. As outlined above, archeocytes are proposed to be totipotent stem cells in demosponges (Funayama 2013). They are characterized by larger size, their large nucleus, and the presence of a single prominent nucleolus, which in freshwater sponges can be seen as a clear area in DNA stains (Simpson 1984; Funayama 2013). In *S. domuncula*, the size of archeocytes has been estimated by electron microscopy to be ∼8 µm in diameter, with nuclei of ∼3 µm in diameter (R. Revilla-i-Domingo, F. Raible, and Alexander Ereskovsky unpublished data). Cells with these features were indeed among the FAC-sorted cells (Figure 4H). While an atlas of molecular signatures of distinct cell types is still missing for *S. domuncula*, the gene *vasa* has been shown to be specifically expressed in the archeocytes of the demosponge *E. fluviatilis* (Alié *et al.* 2015). Molecular phylogeny allowed us to identify an unambiguous *vasa* ortholog in *S. domuncula* (see Figure S5). Using qPCR analysis, we found that *vasa* is not only detectable in the EGFP+ population, but also significantly enriched, when compared to a population of unsorted cells (Figure 4F). Under the assumption that the expression of *vasa* in archeocytes is conserved in *S. domuncula*, these molecular data are consistent with an enrichment of at least one class of putative stem cells in the pool of stably transfected cells.

Taken together, our results outline a robust method for transfecting slice explants of a sponge with exogenous DNA during regeneration, resulting in transcription, faithful splicing, and protein expression in individual cells. The expression of the fluorescent marker protein EGFP allows for reliable recovery of transfected cells, including putative stem cells, from large tissue samples, compatible with an application in transplantation experiments.

## Discussion

Our results extend the toolkit for sponge research in two relevant and complementary ways. First, the slice explant system we established in *S. domuncula* complements existing experimental paradigms for investigating sponge development. Various researchers have explored the asexual reproduction system of some sponges to gain insight into sponge development, by investigating the development of gemmules in culture at the molecular level (for example Rivera *et al.* (2013), Nakayama *et al.* (2015), Windsor Reid *et al.* (2018)). The explant culture we established offers a chance to compare insights from such gemmule-based developmental studies with regeneration processes in adult sponges. As mentioned, *S. domuncula* also produces gemmules (Le Pennec *et al.* 2003), thus offering the possibility to perform such comparisons within the same individual.

A second contribution of this work concerns the tool of transgenesis. To our knowledge, our results represent the first report of successful transgenesis in sponges. We reason that the lack of any previous success might be attributed to differences in the efficiency between transgenesis methods: Whereas transfection with linear polyethyleneimine has allowed us to efficiently introduce exogenous DNA into sponge cells at a dose of 50–110 copies per cell, and to express detectable levels of EGFP, systematic electroporation trials using the same expression construct resulted in at least 100 times lower exogenous DNA introduction, without detectable levels of *egfp* transcript (data not shown). This suggests careful quantification of DNA uptake to be a critical step in devising transgenic strategies in other systems. We further note that polyethyleneimine-based strategies have been shown to successfully deliver exogenous DNA in other marine organisms, where liposome-based delivery reagents, such as lipofectamine, yielded less efficient results (Calderon and Sun 2003; Sun *et al.* 2005). These results may hint at a general difference in efficiency for marine species, possibly linked to salinity conditions.

Despite the low proportion of cells with detectable EGFP fluorescence (∼1 in 7000), the established methodology may already provide a first tool to trace cells in transplantation experiments, paving the way for the study of cellular potency. Determining the potential of stem cells in sponges has so far relied on morphological observations (Simpson 1984; Ereskovsky 2010), coexpression of stem cell and differentiation markers (Funayama 2013), and CM-DiI labeling (Nakanishi *et al.* 2014). While these techniques have been extremely useful for establishing cellular lineages in the sponge (Funayama 2013), transgenic labeling of individual cells holds significant potential for expanding this field, especially in light of the current improvements in single-cell sequencing technology.

For many nonconventional model systems, the establishment of transgenesis has represented a critical bottleneck. Overcoming this bottleneck has subsequently triggered rapid progress in the establishment of functional tools. The advances presented here may therefore help to advance the development of additional functional techniques – such as expression of dominant negative forms of genes, or genome targeting – that will help to push functional research in a cornerstone phylum for evolutionary, developmental, and stem cell biology.
